# Development and Validation of the Policies, Opportunities, Initiatives and Notable Topics (POINTS) Audit for Campuses and Worksites

**DOI:** 10.3390/ijerph16050778

**Published:** 2019-03-04

**Authors:** Tanya M. Horacek, Marlei Simon, Elif Dede Yildirim, Adrienne A. White, Karla P. Shelnutt, Kristin Riggsbee, Melissa D. Olfert, Jesse Stabile Morrell, Anne E. Mathews, Wenjun Zhou, Tandalayo Kidd, Kendra Kattelmann, Geoffrey Greene, Lisa Franzen-Castle, Sarah Colby, Carol Byrd-Bredbenner, Onikia Brown

**Affiliations:** 1 Department of Public Health Food Studies and Nutrition, Syracuse University, Syracuse, NY 13244, USA; mbsimon21@gmail.com; 2 Department of Food and Nutrition, Augusta University Medical Center, Augusta, GA 30904, USA; 3 Human Development and Family Studies, Auburn University, Auburn, AL 36849, USA; elifdy@auburn.edu; 4 School of Food and Agriculture, University of Maine, Orono, ME 04469-5735, USA; awhite@maine.edu; 5 Department of Family, Youth and Community Sciences, University of Florida, Gainesville, FL 32611, USA; kpagan@ufl.edu; 6 Department of Nutrition, University of Tennessee, Knoxville, TN 37996, USA; kolmstea@vols.utk.edu (K.R.); scolby1@utk.edu (S.C.); 7 Division of Animal and Nutritional Sciences, School of Agriculture, West Virginia University, Morgantown, WV 26506, USA; melissa.olfert@mail.wvu.edu; 8 Department of Molecular, Cellular and Biomedical Sciences, University of New Hampshire, Durham, NH 03824, USA; jesse.morrell@unh.edu; 9 Food Science and Human Nutrition Department, University of Florida, Gainesville, FL 32611, USA; anne.mathews@ufl.edu; 10 Department of Business Analytics and Statistics, University of Tennessee, Knoxville, TN 37996, USA; wzhou4@utk.edu; 11 Department of Food, Nutrition, Dietetics and Health, Kansas State University, Manhattan, KS 66506, USA; martan@k-state.edu; 12 Health and Nutritional Sciences Department, South Dakota State University, Brookings, SD 57007, USA; kendra.kattelmann@sdstate.edu; 13 Department of Nutrition and Food Sciences, University of Rhode Island, Kingston, RI 02881, USA; ggreene@uri.edu; 14 Department of Nutrition and Health Sciences, University of Nebraska-Lincoln, Lincoln, NE 68588, USA; lfranzen2@unl.edu; 15 Department of Nutritional Sciences, Rutgers University, New Brunswick, NJ 08901, USA; Bredbenner@sebs.rutgers.edu; 16 Department of Nutrition, Dietetics and Hospitality Management, Auburn University, Auburn, AL 36849, USA; onb0001@auburn.edu

**Keywords:** environmental audit, web-based assessment, health promotion policy, college

## Abstract

*Background:* Workplace or campus wellness/obesity-prevention policies and initiatives can improve health. Research tools to assess worksite or campus policies/initiatives are scarce. Thus, the aim of this research is to develop and validate the policies, opportunities, initiatives, and notable topics (POINTS) audit. *Methods:* POINTS was developed and refined via expert review, pilot-testing, and field testing. Trained researchers completed a web-based review from a student-focus or employee-focus regarding 34 health-promoting topics for colleges. Each topic was evaluated on a 0–2 scale: 0 = no policy/initiative, 1 = initiatives, 2 = written policy. When a written policy was detected, additional policy support questions (administered, monitored, reviewed) were completed. *Results:* Cronbach’s Alpha for the student-focused POINTS audit was *α* = 0.787 (34 items, possible points = 65), and for the employee-focused POINTS audit was *α* = 0.807 (26 items, possible points = 50). A total of 115 student-focused and 33 employee-focused audits were completed. Although there was little evidence of policy presence beyond stimulant standards (smoking and alcohol), there were extensive examples of health initiatives. The student-focused POINTS audit was validated using the Healthier Campus Initiative’s survey. *Conclusions:* POINTS is a web-based audit tool that is valid and useful for pre-assessment, advocacy, benchmarking, and tracking policies for health and well-being for students (campus) and employees (worksite).

## 1. Introduction

Over one-third of adults in the United States are obese [1]. Researchers have shown environmental factors influence weight status [2,3,4]. Unfortunately, much of the current evidence for the college environment suggests that both students and employees default to sedentary and poor dietary intake behaviors [5,6,7,8]. Further, other studies have found that healthy work environment initiatives can improve employee wellness and reduce employer health-related expenses [9,10]. Initiatives are interventions or programs intended to encourage healthy behaviors and decisions. Whereas, a policy is a written and published document outlining a definite course or method of action to determine and guide present and future decisions. Policies are most effective if they have defined goals and procedures for implementation, including a charged department or individual responsible for their implementation. Policies promoting a healthy workplace may improve dietary intake, decrease sedentary behavior, and increase overall health-promoting behaviors [10,11,12].

Colleges as a workplace are required to mandate employee policies regarding overtime, medical leave, and occupational health and safety [13], and are audited by state and sometimes federal agencies. The Center for Disease Control and Prevention urges employers to implement health promotion policies in the workplace [14]. Numerous resources and best practice recommendations for workplace health and wellness policies are available for employers through federal, state, and other agencies [15,16,17,18]. However, nutrition and wellness policies are only mandated in the public-school system [19,20,21]. An extensive body of literature exists regarding the evaluation of school wellness policies [16,22,23,24,25,26,27] and many of these policy evaluation tools, although comprehensive [16,23,25,26], are tailored for the elementary or high school environment. 

Tools for assessing worksite or college policies/initiatives are lacking. Using a 21-question yes/no survey, one study assessed worksite healthy supports and policies [28]. However, only two of the 21 questions were about policies, the remaining items were environmental supports or interventions. On an international level, another study conducted semi-structured interviews with key stakeholders to evaluate the extent to which nutrition topics and policies were implemented [29]. The study authors developed and used a policy assessment tool based upon the “four Ps” marketing approach (price, product, place, and promotion) for health or nutrition policy. The researchers concluded that mandatory (policies/laws) versus voluntary initiatives were more effective for improving health yet less obvious.

Likewise, the American College of Health Association encourages colleges to set and track effective health goals for their campuses [30]. The Partnership for a Healthier America, specifically the Healthier Campus Initiative (HCI) [31], established 41 guidelines, with the criteria that campuses meet at least 23 of the 41 guidelines to be designated a Healthier Campus. However, currently there is no tool designed to assess the extent to which wellness and obesity-prevention policies, in general, are implemented for college campuses. The HCI lists specific policies and initiatives with a yes/no evaluation, which fails to evaluate other examples of policies or the level of policy integration or support. The college campus is a unique environment in that it serves as both a learning institution and workplace. Some college campuses are similar in size to a city/village and are typically one of the largest employers in many communities. A policy audit tool appropriate for this environment may also be effective in a variety of similar educational/work settings. Well implemented wellness and obesity prevention policies and initiatives can greatly improve health habits of both students and employees [27,32].

Tools exist to evaluate wellness policies in public schools and government entities; however, no tool exists to evaluate wellness policies or initiatives for educational workplaces or college settings. The purpose of this paper is to describe the development, field testing, and validation of the policies, opportunities, initiatives, and notable topics (POINTS) audit for college campuses and worksites.

## 2. Methods

### 2.1. Overview

This paper describes the two phases used in the development of the POINTS. For phase one, instrument development, the audit was developed using a three-step process: (1) Inventory item development; (2) expert, pre- and pilot-testing, and audit revisions; and (3) field implementation. For phase two, instrument validation, POINTS was validated using the Healthier Campus Initiative’s (HCI’s) [31] 41 guidelines. Data were collected between 2015–2017 and analyzed in 2018. Syracuse University’s Institutional Review Board determined this research to be exempt because this was environmental, not human research.

### 2.2. Methods: Instrument Development (Phase One)

#### 2.2.1. Development of Inventory Items for the Audit

To develop the audit, the authors completed a thorough review of the literature and health expert policy recommendations. In addition to the peer-reviewed literature, the authors searched online and considered workplace and school wellness recommendations made by government agencies, non-profit health organizations, and health professionals. For purposes of this audit, the following definitions clarify policy, initiative, and a pledge:

- **Policy**: A written and published document outlining a definite course or method of action to determine and guide present and future decisions. Policies may have defined goals and procedures for implementation, including a charged department or individual responsible for their implementation.
- **Initiative**: A series of interventions or programs intended to encourage healthy behaviors and values. Initiatives may or may not contain any defined goals, procedures, or plans for implementation.
- **Pledge/Commitment**: A written and published agreement that is not specifically designated as being a policy. Pledges/commitments may or may not have defined goals or procedures for implementation.

Thirty-four health, wellness, obesity-prevention, and sustainability topics were extracted from the literature review. The 34 topics (Table 1; to see the wording of each question, refer to Supplementary Materials S1 were content analyzed and grouped into the seven categories accordingly:

1. Stimulant Standards: Smoking independently increases the risk of cardiovascular disease [33]. Excessive alcohol consumption increases the risk of weight gain [34], and other deleterious outcomes for students [35,36]. Randomized control trials found positive results for workplace wellness initiatives and environmental supports that encourage smoking cessation [37,38].
2. Chronic Disease Management and Health Promotion: Numerous studies including health, nutrition, and/or physical activity education improved health outcomes [39,40,41]. Worksites with healthy environmental policies and initiatives reduced medical costs and increased savings [9].
3. Healthy Student Course Requirement: Health and nutrition education programs effectively increased college students’ physical activity, fruit/vegetable intake [42,43], and their overall knowledge about nutrition [44].
4. Health and Wellness Services: Workplaces that have wellness departments or professionals were more likely to have wellness programs and policies. [45]. Employees were more likely to participate in physical activity and make healthier choices if they were incentivized (i.e., rewards/prizes or lower health insurance premium rates) [46,47].
5. Active Living: Employees were more likely to partake in non-work physical activity in safe and well-maintained environment (i.e., sidewalks and stairwells) [39,48,49,50,51]. Soler and colleagues encouraged workplaces to adopt numerous environmental policies which support safe environments and encourage physical activity [52]. The growing concern over carbon emissions has also motivated policy makers and key stakeholders to create and implement policies that discourage driving and encourage walking or biking to work [53,54].
6. Nutritious and Sustainable Food Ways: Strong evidence exists regarding the relationship between the food environment and healthy eating patterns [55,56]. Behavioral economics and nutrition food policies can reduce obesity and positively influence dietary habits [57,58,59,60]. National nutrition policies for public schools receiving federal funding have been required for decades, and some states now have nutrition menu labeling policies. There are limited healthy choices in restaurants, stores, and vending machines on- and near-college campuses [61,62], so access to a farmer’s market [63,64] and food procurement policies on campus might positively effect non-communicable disease risk [65,66,67]. Sustainability is of growing concern on campuses and can have both health and environmental benefits [53,68,69].
7. Healthy Student Living: Some environmental research suggests a health benefit to living on campus as compared to living off campus [70,71], so the policies for on-campus housing, dining hall contracts, and food security initiatives are important to review.

Although the audit is tailored primarily for the student population, as we reviewed the literature we noted the topics unique to a student versus employee population. Using skip logic, the audit can be used to evaluate the existence and extensiveness of policies affecting the employee population. Table 1 indicates which topics are included in each audit version.

**Figure 1:**
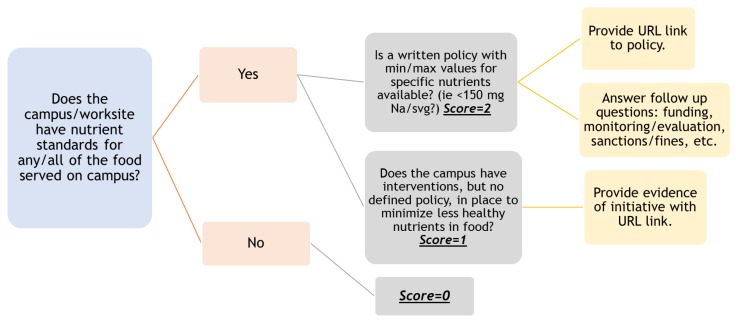
An example of the phases of identifying a policy with POINTS, regarding nutrient standards.

Each audit question was scored on a three-point semantic differential scale to assess each policy topic (0 = no policy; 1 = initiative/interventions; 2 = written policy) [16,23]. When a policy was identified, the written policies were further assessed for the total comprehensiveness of the policy including: defined mission/goals, policy outcomes, implementation plan, a department charged with implementation, defined sanctions/fines for policy violation, monitoring/evaluation plan, and policy review plan. Additionally, when a policy was identified, the evaluator submitted a copy of the policy (via an URL link) on the Qualtrics survey. See Figure 1 for an example.

#### 2.2.2. Expert, Cognitive, and Pilot Testing

POINTS was reviewed by ten experts in nutrition, health promotion, physical activity, and public health from various institutions to establish content validity. The POINTS audit was also cognitively tested with five research assistants to ensure the items were interpreted accurately. Cognitive testing and expert review resulted in improved wording of questions and semantic-differential response choices.

For the pre-test, the lead authors interviewed three wellness and obesity-prevention professionals at a university located in the northeast regarding the campus health and wellness policies. Interviews were conducted in summer 2015. The professionals answered open-ended and non-leading questions such as “What policies exist on campus regarding food nutrient standards for the campus population?” The professionals were unable to identify the difference between a policy and initiatives. Monitoring and evaluating the outcomes of initiatives and policies were scarce.

After refinements, POINTS was developed into the online survey for health promotion professionals. This survey was pilot-tested in fall 2015 at 15 US college campuses. The authors identified 51 wellness and obesity-prevention professionals within their universities to be contacted (one to four per campus). All 51 professionals were contacted via telephone by undergraduate/graduate student research assistants. The professionals were given a brief explanation of the survey and invited to partake in the research. If they agreed to participate, they were sent a website link to the survey via email. The professionals were directed to only complete questions that pertained to their job title and duties (e.g., foodservice manager—Nutrition and Sustainable Food Ways section) but often each professional completed the whole survey. When the discrepancy was detected, we reviewed their policies via a web-search to determine correctness of responses. Since professionals answered from their own perspective, without doing any additional research to verify their answers, there was very little agreement between different health promotion experts on a campus. Because of these limitations in the professional survey, the research team decided the audit should be completed as a web-review by trained research assistants.

#### 2.2.3. Field Testing Training and Interrater Reliability (IRR)

Data were collected in spring 2016 through spring 2017 at a total of 115 campuses. Campuses (*n* = 80) participating in the Get FRUVED [72] project collected the student-oriented POINTS audit as part of their participation in the social marketing and environmental intervention. Thirty-five additional campuses were identified and evaluated by the lead institution’s research assistants for both the student-focus and employee-focus audits. Researchers were trained to complete web searches to identify policy statements using the three-point semantic differential system. Data were collected through online survey software Qualtrics ™ (Qualtrics, Seattle, WA, USA) and proof of policy was the submission of the webpage URL links.

Training and interrater reliability (IRR): Research assistants completed online video-based training that taught them how to: (1) Prepare for a successful audit; and (2) interpret and answer each audit question with respect to the varied web environments. Then, they practiced using the POINTS audit on two different school websites. Subsequently, they independently used the POINTS audit to evaluate two new university campuses, which were not included in practice sessions, to establish IRR. The data were compared to the standard set by the lead institution. POINTS audits were repeated until all data collectors on a campus achieved an IRR > 0.80, before they commenced with data collection. As more independent schools joined the data collection in early 2017, the IRR protocol was changed to an online quiz.

### 2.3. Data Analysis

In addition to a total POINTS score, sub-scores were created for each of the categories on the audit: Stimulant Standards, Chronic Disease and Health Promotion, Healthy Student Required Classes, Health and Wellness Services, Active Living, Nutritious and Sustainable Food Ways, and Healthy Student Living. The policy support score was the total comprehensiveness of the policies; the summation of the eight follow up questions when a policy existed—defined mission/goals, policy outcomes, implementation plan, a department charged with implementation, defined sanctions/fines for policy violation, monitoring/evaluation plan, and policy review plan. SPSS (version 24, IBM, Armonk, NY, USA) was used to run non-parametric statistics, t-tests, and ANOVA. Level of significance was set at *p* < 0.05.

### 2.4. Results for Instrument Development

A total of 115 student-focused and 33 employee-focused audits were collected by trained student research assistants, who had satisfactory IRR (*α* = 0.783). The mean time to collect and enter the data was 3.75 ± 3.6 h; median 2.5 h. Cronbach’s Alpha for the student-focused POINTS audit was *α* = 0.787 (35 items, total potential points = 65), and for the employee-focused POINTS audit was *α* = 0.807 (26 items, total potential points = 50). More of the audits were collected from public institutions for both the student- and employee-focused audits (85% and 63.6%, respectively) (Table 2). For the student-focused audit, the highest percentage of audits were collected from the southeast region (40%), followed by the midwest and northeast; the least were collected from the southwest (3.5%). For the employee-focused audits, the geographic distribution was similar, whereas the smallest percentage of audits was collected from the northwest (6.1%).

Average student population was 18,952 for the student-focused POINTS audit, with the employee-focused audits slightly larger at 21,297 students. Based on the distribution of campuses by size, schools were grouped by student population size. Very small campuses had a student population <4500 students. Small schools had 4501 to 12,500 students. Midsized schools were defined by a population of 12,501 to 17,500. Large schools had 17,501 to 29,000 students, and very-large schools had >29,001 students. School characteristics data are listed in Table 2.

For the student-focused POINTS audit, almost all campuses (at least 90%) had smoking and alcohol/substance abuse policies (Table 3). Dining hall contracts and on-campus living policies were in place for a moderate percentage of schools (65.2% and 50.4%, respectively). Policy presence evidence was detected for health and wellness departments (37.4%), insurance premium incentives (29.6%), designated eating environments (17.4%), healthy campus fund raising (15.7%), and health education for credit (12.2%). The remaining 22 topics had less than 10% of schools with evidence of policy presence; however, at least 75% of the schools had intervention presence for 13 of the topics (non-credit health, nutrition or physical education; health screenings, environmental supports for active living, closed campus, sustainable transportation, healthy food options, local and sustainable food, organic waste reduction and disposal, farmer’s markets, campus garden, and an open campus).

For the employee-focused POINTS audit, at least 93% of the campuses had smoking and alcohol/substance abuse policies (Table 3). Additional policy presence evidence was detected only for insurance premium incentives (27.3%) and health and wellness departments (12.1%). The remaining 20 topics had only one–two schools with evidence of policy presence; however, at least 75% of the schools had intervention presence for 15 of the topics (non-credit health, nutrition or physical education, health habit challenge, health and wellness services, environmental supports for active living, closed campus, sustainable transportation, healthy food options, designated eating environment, local and sustainable food, organic waste reduction and disposal, and farmer’s markets).

The Total POINTS score for student-focused audits indicated more interventions than policies with a mean 30.13 ± 5.48, maximum possible 65; and low overall policy support with a mean of 23.2 ± 12.2, maximum possible 320) (Table 4). Sub-score means are an indication of the total number of interventions and policies present for each of the sub-categories. The Stimulant Standards had the highest evidence for policy/intervention presence (3.8 ± 0.5; max possible 4) and policy support (8.1 ± 3.6; max possible 16). Healthy Student Living followed closely behind (4.51 ± 1.01; max possible 7), but had significantly less policy support (4.0 ± 4.1, max possible 24). Health and wellness services on campuses were supported by a balance of policies and interventions (2.92 ± 1.31, maximum possible 6) and reasonable professional and policy support (7.4 ± 5.2, max possible 32). Although few policies existed for individual Nutritious and Sustainable Food Ways (Table 3), the mean score for campuses conveyed the high degree of interventions in place (7.46 ± 2.01, max possible 18) and a lack of policy support. The remaining three sub-categories: Chronic Disease and Health Promotion, Healthy Student Requirements, and Active Living had lower policy/intervention evidence and consequently very low policy support. A few significant differences were evident based upon demographic variables. Private schools as compared to public had significantly less evidence for policy presence for Active Living (2.57 ± 0.86 vs. 3.09 ± 1.10, *p* < 0.01); and Chronic Disease and Health Promotion (5.20 ± 2.35 vs. 6.46 ± 2.03; *p* < 0.01). Although there were no differences in total score or sub-score by geographic region, differences were detected by campus size. The smallest schools’ means were significantly lower than all school categories for Chronic Disease and Health Promotion (very Small schools: 4.09 ± 0.58 vs. (small: 6.0 ± 0.47, to 7.13 ± 0.30 for very large schools) *p* < 0.01. The smallest schools had scores significantly lower than all schools larger than 12,501 students on Active Living (very small: 2.24 ± 0.32 vs. (moderate sized schools: 3.10 ± 0.17 to 3.46 ± 0.17 for very large schools) *p* < 0.01 and on total POINTS, very small schools: 26.67 ± 1.68 vs. (Moderate sized schools: 30.76 ± 0.82 to 32.8 ± 0.85 for very large schools), *p* < 0.01).

There was an equal split between the universities with contracted food service departments (*n* = 58) and those independently run by their campus (*n* = 57). Although there was no significant difference in the total Nutritious and Sustainable Food Ways subscale total, contracted departments had significantly more policy support than independently run systems (2.51 ± 3.9 vs. 0.94 ± 1.73 respectively; *p* = 0.006.

The Total POINTS and sub-category scores for the employee-focused audits indicated very similar results to the student-focused audits (Table 4). The employee-focused POINTS audit was significantly lower for private institutions as compared to public, and had significantly less evidence for policy presence for Chronic Disease and Health Promotion (5.08 ± 2.50 vs. 7.00 ± 1.92, *p* < 0.05); Employee Health and Wellness (1.91 ± 0.51 vs. 2.48 ± 0.68; *p* < 0.05) and total POINTS (20.67 ± 4.92 vs. 25.10 ± 3.42; *p* < 0.05). There were no significant differences by campus size or geographic region.

## 3. Results

### 3.1. Methods for Validation of POINTS (Phase Two)

In collaboration with the Partnership for Healthier America’s Healthier Campus Initiative (HCI) [31], participating institutions (*n* = 60) in Get FRUVED [72] completed both POINTS and the HCI survey. The HCI survey was created for this study based upon the HCI guidelines and was chosen for validation, because it measures comparable concepts for the college campus. All audits completed were for the student-focused population.

#### Comparison Tool

The HCI survey contained 41 questions, 15 regarding food and nutrition, 19 regarding physical activity, and seven regarding programming. Programming topics were similar to the POINTS Chronic Disease and Health Promotion category. Each question was a Yes/No check off to indicate if a campus had the initiative or policy. A summary of the topics assessed are listed in Table 5, with a more detailed listing of the questions included in Supplementary Materials S2.

### 3.2. Data Analysis

Each HCI category and the total scores were tallied. Spearman’s correlations were run on POINTS and HCI survey totals and sub-scores.

### 3.3. Results for the Validation Study

Fifty-six of the 60 Get FRUVED intervention schools had matched POINTS and HCI data. Most of the schools were public institutions (76.8%) (see Table 6). The southeast represented 41.1% of the sample, while only 3% were from the southwest. Half the sample (50%) were from smaller schools with ≤ 12,500 students.

There were significant correlations between total POINTS and the HCI total (*r* = 0.519, *p* < 0.01), and total Policy Support and the HCI total (*r* = 0.478, *p* < 0.001), as well as between Nutritious and Sustainable Food Ways (POINTS) and the Food and Nutrition HCI sub-score (*r* = 0.314, *p* = 0.019). There were no significant correlations between POINTS for Chronic Disease Health Promotion and Programming (*r* = 0.101, *p* = 0.46) or between both tools for Physical Activity—Active Environment (*r* = 0.210, *p* = 0.12).

## 4. Discussion

As a result of this research a new tool was produced to assess the extent of health and wellness policies and initiatives on college campuses supporting students’ and employees’ health. Through a web review of policies and interventions, student research assistants were able to reliably complete student-focused or employee-focused audits. The tool provides a simple total score and sub-scores for ease of interpretation. In general, college campuses lack policy support for health and wellness-related areas beyond smoking and alcohol. POINTS was designed to capture what health promotion/disease prevention policies should be on record, so one would not expect a significant number of institutions to have an extensive list of these policies implemented. There were some differences noted by campus size and public/private status. It was encouraging to note the extensiveness of the interventions in place, but to make them sustainable and enforceable, policy support must be implemented (mission, enforcement, and monitoring). POINTS was also validated by experts, and for the student-focused audit, by comparison to the HCI-recommended guidelines.

Policy evaluation tools exist for schools, day care centers [16,23,73], and communities [25,26,29], but not for worksites/universities. The POINTS tool is easy to use and objective. It is conducted by searching for evidence of policy/initiative presence online. With training, students effectively implemented this protocol. The average time for data collection was 3.75 hours per institution, but the median was 2.5 hours. Differences in website design and evaluator approach to the task would lead to wide range effort.

To the authors’ knowledge, comparisons in the literature for this study do not exist. Much of the literature regarding policy research has been with school districts, not college campuses or worksites. In those studies, reviews were completed to determine if school wellness policies existed [22,74]. There was one report of an intervention to improve the enforcement of a policy (i.e., after-school snack policy) [75], implying sometimes policies need intervention support to verify their need/effectiveness. Some studies found more policy support in places for where there was a higher need (school districts with higher free lunch participation and/or obesity) [76,77]. Many times, the papers focused on factors affecting their ability to implement policies [19,74,75,77].

Policies can make a difference. For all Minnesota school districts, more comprehensive physical activity policies were associated with greater exercise [76]. States with stronger laws restricting advertising and competitive foods/beverages sales had reduced incidence of obesity [27]. In another study, restricting sugar-sweetened beverages was most effective for improving milk intake [78].

Based upon a systematic review for worksites, there has been limited evidence for the effectiveness for worksite environmental interventions and very few policies on record to support healthful behaviors [51]. However, in more recent studies, healthy work environment interventions improved employee health and reduced employer health-related expenses [9,10]. Health risk assessments with feedback and education have proven to be effective for improving a variety of health parameters [41] and; therefore, should be considered as a policy for worksite environments.

For either a student-focused or employee-focused environment, prior research provides some guidance regarding effective policy efforts. Dodson and colleagues [39] found the more structured and specific the worksite policies, the more likely the employees were to achieve exercise recommendations. Similarly, college campuses might require physical (or nutrition/health) education classes. In this study, there were less than 15% of the schools that had such policies. Historically these classes were required on more campuses but the trend for such requirements has been declining [79,80,81]. Other studies provide evidence that nutrition environment policies for catering and point of purchase labeling affect behavior [55] and taxing incentives (subsidy for healthy and tax for unhealthy) might be effective and affect predicted buying behavior and nutrient quality [56].

The POINTS research found smaller campuses had lower policy evidence and supports. Similarly, Brissette and colleagues [28] found of the 832 worksites evaluated, smaller companies had less cardiovascular health policies. When a company indicated a wellness committee/coordinator, more policies were evident, indicating the importance of staffing supports for policy implementation.

There is a need for more accuracy and consistent definitions regarding policies versus initiatives. Interventions and initiatives may be effective resulting in appropriate behavior change, but without policy support, they are often temporary and fleeting. Even with policy support, one study evaluating the effectiveness of school wellness policies and practice found very low agreement between the written policy and nutrition-related practices [24]. Having a policy on record is just the first step. The degree of policy support, specifically how well it is managed, enforced, monitored, and reviewed are important determinants of effectiveness. In a study of the Minnesota school districts [76], those with higher levels of poverty and obesity implemented higher quality school wellness policies in terms of strength and comprehensiveness. Reviewing the history for smoking policies, we can trace the successes and challenges with policy implementation. While Hopkin and colleagues [37] found effective policies reduced tobacco use, based on a systematic review, weak evidence was secured for the effectiveness of strategies for enforcing smoke-free policies [82]. Best practices for implementing policies include securing administrative buy-in, relationship building, conveying effectiveness, and conveying financial sustainability [83,84].

The strength of the POINTS tool is that the policy items assess the health promotion and wellness concepts that should be implemented in worksites and on college campuses. In addition, a clear distinction is made between policies and interventions/initiatives, and it is specific yet flexible enough to be effective for a variety of student and employee populations. The POINTS audit and an extensive training with an IRR quiz are online. The user is provided with feedback and comparative results. Users can pre-assess their policies and interventions, advocate for changes, and track their progress over time.

A limitation is the small sample size and the disproportionate representation by campus size/geographic location. Additionally, student research assistants implementing this internet-based audit might lack access to some policies which might be stored on a campus intra-net system. Finally, although the student-focused audit was validated against the HCI and had moderate correlations for the total and the food/nutrition scores, there were no correlations for the physical activity or health promotion categories. Future studies need to compare POINTS to student and employee health data and, validate the employee-focused POINTS audit against another tool, and with a diversity of campus and work environments.

## 5. Conclusions

POINTS is a web-based audit tool that is valid and useful for pre-assessment, advocacy, benchmarking, and tracking policies for health and well-being for students and employees. The results of this study should act as motivation to implement high-quality health and wellness policies on campuses and worksites, as this tool provides a way to monitor progress and improvement.

## Figures and Tables

**Table 1:** Categories and topics assessed via the policies, opportunities, initiatives, and notable topics (POINTS) audit ^1^.

POINTS Categories	POINTS Sub-Categories	Specific Topics
Stimulant Standards [37,38]		Smoking Alcohol
Chronic Disease Prevention	Chronic Disease Management and Health Promotion [9,39,40,41]	Health education not for credit Nutrition education not for credit Physical education not for credit Health promotion—all forms of media Health fairs Health screenings Chronic disease education Health habit challenges
	Healthy Student Course Requirements [42,43,44] ^2^	Health education for credit Nutrition education for credit Physical education for credit
	Health and Wellness Services [45,46,47]	Campus health and wellness department Healthy campus fundraising ^2^ Healthy employee insurance premiums
Active Living [39,48,49,50,51,52]		Physical activity during work hours Active environments (i.e., bike lanes, stairs) Closed campus Sustainable transportation
Nutrition Environment	Nutritious and Sustainable Food Ways [51,55,56]	Healthy food options Nutrient standards Healthy food labels and point-of-purchase nutrition info Food taxes and subsidies Designated eating environments Local and sustainable food Organic waste reduction and disposal Farmers markets Local food access on-campus Campus food gardens
	Healthy Student Living [70,71] ^2^	On-campus housing Open campus Dining hall contracts Food security initiatives

^1^ To see the wording of each question, refer to Supplementary Materials S1. ^2^ Questions factored only into the student version of the POINTS audit.

**Table 2:** Characteristics of schools with completed POINTS audits.

Variables	Student-Focused Audit *n* = 115	Employee-Focused Audit *n* = 33
Institution Classification	% (*n*)	% (*n*)
Private	26.1 (30)	36.4 (12)
Public	73.9 (85)	63.6 (21)
Geographic Location		
Midwest	18.2 (21)	18.2 (6)
Northcentral-Midwest	11.3 (13)	15.2 (5)
Northeast	17.4 (20)	21.2 (7)
Northwest	9.6 (11)	6.1 (2)
Southeast	40.0 (46)	30.3 (10)
Southwest	3.5 (4)	9.1 (3)
School Size—Student Population	18,952 ± 15,012	21,297 ± 16,827
School Size Range	(367 to 68,942)	(1136 to 68,942)
Very Small: <4500	18.3% (21)	15.2% (5)
Small: 4501 to 12,500	21.7% (25)	15.2% (5)
Moderate: 12,501 to 17,500	18.3% (21)	21.2% (7)
Large: 17,501 to 29,000	20.9% (24)	15.2% (5)
Very Large: >29,001	20.9% (24)	33.3 % (11)
Employee Population	3382 ± 5245	3933 ± 6474
Employee Population Range	(9 to 22,000)	(120 to 22,000)

**Table 3:** Frequency of policy and intervention presence for the student-focused and employee-focused POINTS audits.

Sub-Categories	Specific Topic	Student-Focused Audit		Employee-Focused Audit	
		Policy	Intervention	Policy	Intervention
		% (*n*)	% (*n*)	% (*n*)	% (*n*)
Stimulant Standards	Smoking	90.4 (104)	8.7 (10)	93.9(31)	6.1(2)
	Alcohol	91.3 (105)	7.0 (8)	97 (32)	0
Chronic Disease and Health Promotion	Health education non-credit	3.5 (4)	76.5 (88)	3 (1)	75.8 (25)
	Nutrition education non-credit	1.7 (2)	80.9 (93)	0	81.8 (27)
	Physical education non-credit	0.9 (1)	87.0 (100)	3 (1)	84.8 (28)
	Health promotion media	4.3 (5)	72.2 (83)	3 (1)	78.8 (26)
	Health fairs	1.7 (2)	67.8 (78)	3 (1)	60.6 (20)
	Health screenings	2.6 (3)	79.1 (91)	3 (1)	72.7 (24)
	Chronic disease education	1.7 (2)	47.0 (54)	0	63.6 (21)
	Health habit challenges	0.9 (1)	67.8 (78)	0	81.8 (27)
Healthy Student Course Requirements	Health education for credit ^1^	12.2 (14)	56.5 (65)	NA	NA
	Nutrition education for credit ^1^	6.1 (7)	61.7 (71)	NA	NA
	Physical education for credit ^1^	8.7 (10)	63.5 (73)	NA	NA
Health and Wellness Services	Health and wellness dept.	37.4 (43)	56.5 (65)	12.1 (4)	78.8 (26)
	Healthy campus fundraising ^1^	15.7 (18)	18.3 (21)	NA	NA
	Insurance premium incentives	29.6 (34)	52.2 (60)	27.3 (9)	69.7 (23)
Active Living	Physical activity during work	6.1 (7)	20.9 (24)	6.1 (2)	72.7 (24)
	Environment supports (i.e., bike lanes, stairs)	6.1 (7)	76.5 (88)	3 (1)	81.8 (27)
	Closed campus	2.6 (3)	80.0 (92)		93.9 (31)
	Sustainable transportation	6.1 (7)	76.5 (88)	3 (1)	87.9 (29)
Nutritious and Sustainable Food Ways	Healthy food options	4.3 (5)	85.2 (98)	3 (1)	93.9 (31)
	Nutrient standards	0.9 (1)	47.8 (55)	3 (1)	39.4 (13)
	Healthy food labels and point-of purchase nutrition info	4.3 (5)	58.3 (67)	3 (1)	63.6 (21)
	Food taxes and subsidies	2.6 (3)	0	0	0
	Designated eating environments	17.4 (20)	66.1 (76)	3 (1)	75.8 (25)
	Local and sustainable food	1.7 (2)	80.0 (92)	3.0 (1)	84.8 (28)
	Organic waste reduction and disposal	7.0 (8)	80.9 (93)	6.1(2)	81.8 (27)
	Farmers markets (% yes)	NA	96.5 (111)	NA	100
	Local food access on-campus	2.6 (3)	72.2 (83)	0	90.9 (30)
	Campus food gardens (% yes)	NA	77.4 (89)	NA	69.7 (23)
Healthy Student Living	On-campus housing ^1^	50.4 (58)	47.0 (54)	NA	NA
	Open campus (% yes) ^1^	NA	93.0 (107)	NA	NA
	Dining hall contracts ^1^	65.2 (75)	29.6 (34)	NA	NA
	Food security initiatives ^1^	0.9 (1)	48.7(56)	NA	NA

^1^ Questions added only into the student-focused version of the POINTS audit. NA: This question is Not Applicable to the employee population.

**Table 4:** Mean sub-score, total, and policy support POINTS scores.

Student-Focused Audit (*n* = 115)	Policy/Intervention Presence			Policy Support		
	Mean ± SD	Range	Max	Mean ± SD	Range	Max
Stimulant Standards	3.8 ± 0.5	(1,4)	4	8.1 ± 3.6	(0,16)	16
Chronic Disease and Health Promotion	6.1 ± 2.2 ^1,2a^	(0,11)	16	0.7 ± 2.3	(0,17)	64
Healthy Student Required Classes	2.4 ± 1.3	(0,6)	6	0.8 ± 2.1	(0,12)	24
Health and Wellness Services ^3^	2.9 ± 1.3	(0,6)	6	7.4 ± 5.2	(0,27)	32
Active Living	2.9 ± 1.1 ^1,2b^	(0,7)	8	0.6 ± 1.7	(0,10)	32
Nutritious and Sustainable Food Ways	7.5 ± 2.0	(2,13)	18	1.7 ± 3.1	(0,20)	128
Healthy Student Living	4.5 ± 1.0	(2,6)	7	4.0 ± 4.1	(0,16)	24
Total POINTS	30.1 ± 5.5 ^1,2b^	(12,43)	65	23.2 ± 12.2	(3,62)	320
Employee-focused Audits (*n* = 33)						
Stimulants	3.9 ± 0.4	(2,4)	4	8.8 ± 3.3	(2,16)	16
Chronic Disease and Health Promotion	6.3 ± 2.3 ^4^	(1,12)	16	0.4 ± 2.4	(0,14)	64
Health and Wellness Services	2.3 ± 0.7 ^4^	(1,4)	4	4.6 ± 4.3	(0,22)	16
Active Living	3.6 ± 1.1	(0,6)	8	0.5 ± 1.9	(0,10)	32
Nutritious and Sustainable Food Ways	7.4 ± 1.6	(4,13)	18	1.3 ± 3.6	(0,20)	128
Total POINTS	23.5 ± 4.5 ^4^	(9,32)	50	15.7 ± 9.3	(0,45)	256

^1^ Public institutions scored significantly higher than private institutions, *p* < 0.01. ^2^ Scores are significantly different by school size, *p* < 0.01; ^2a^ Very small schools < 4500 students scored lower than all school size categories; ^2b^ Very small schools < 4500 students scored lower than all schools with > 12,501 students. ^3^ For Health and Wellness Services: The number of health professionals indicated is added into policy support. ^4^ Public institutions scored significantly higher than private institutions, *p* < 0.05.

**Table 5:** Healthy Campus Initiative’s (HCI’s) survey topics.

HCI Category	Topics Assessed
Food and Nutrition	offer wellness meals sufficient whole foods (dining and catering) healthier desserts sufficient healthy beverages (dining and catering) plant-based foods tray-less system provide healthy food labels healthier vending and catering free water Registered Dietitian Nutritionist assessments/counseling limitations on fried foods implement local procurement
Physical Activity	offer bike share/rental sufficient fitness/intramural opportunities monthly intro to movement classes, at least one 15 min physical activity break each day fitness orientations sufficient outdoor activities rental for outdoor equipment sufficient outdoor recreation clinics/trips provide marked walking routes pedestrian crossing sufficient bicycle parking spaces sufficient free access to fitness/recreation center dedicated physical activity space outdoor running/walking track outdoor fitness system certified personal trainers implement bicycle/pedestrian accommodation policy public transportation incentives
Programming	implement integrated/comprehensive wellness program mandatory health and wellness education food insecurity program/policy breastfeeding program/policy health/wellness service learning opportunities offer rewards or rebates for insurance premiums healthy cooking classes

**Table 6:** Characteristics of schools participating in the validation study.

School Characteristics	Frequency % (*n*)
Private	23.2% (13)
Public	76.8% (43)
Geography	
Midwest	12.5 % (7)
North Central Midwest	12.5% (7)
Northeast	16.1% (9)
Northwest	14.3% (8)
Southeast	41.1% (23)
Southwest	3.6% (2)
Campus Size (student enrolment)	
Very Small: <4500	28.6% (16)
Small: 4501 to 12,500	21.4% (12)
Moderate: 12,501 to 17,500	14.3% (8)
Large: 17,501 to 29,000	19.6% (11)
Very Large: > 29,001	16.1% (9)

## Supplementary Materials

The following are available online at https://www.mdpi.com/1660-4601/16/5/778/s1, S1: POINTS audit questions, S2: healthier campus initiative survey questions.
