# Supplementary material for: Development and Validation of the Policies, Opportunities, Initiatives and Notable Topics (POINTS) Audit for Campuses and Worksites
Source: Int J Environ Res Public Health. 2019 Mar 4;16(5):778. doi: 10.3390/ijerph16050778 (PMC6427413; doi:10.3390/ijerph16050778)
Supplement: Supplementary file 1 [file ijerph-16-00778-s001.zip › S1 Policy_POINTS Questions.pdf]

S1: POINTS Audit Questions

The Healthy Environment POINTS Audit evaluates the extensiveness and quality of health promotion and obesity prevention policies and interventions/pledges/initiatives for a campus environment. To successfully complete this audit you need to critically think about the difference between a written policy and an intervention/pledge/initiative.

Policy: a written and published document outlining a definite course or method of action to determine and guide present and future decisions. Policies may have defined goals and procedures for implementation, including a charged department or individual responsible for their implementation.
Initiative: a series of projects or programs intended to encourage healthy behaviors and values. Initiatives may or may not contain any defined goals, procedures, or plans for implementing initiatives.
Pledge/commitment: a written and published agreement that is NOT specifically designated as being a policy. Pledges/commitments may or may not have defined goals or procedures for implementation.

Each of the policy topics follows. When you identify a policy for a specific audit topic, you must also indicate if the policy contains any of the following information:

a. Regarding this policy/pledge (Select all that apply)

It defines a mission or vision.

It defines specific goals or aims.

It defines specific outcomes.

It outlines a plan for implementation.

It charges a department or individual to implement it.

It defines sanctions and fines.

It defines a plan for monitoring and evaluating.

It defines a plan for reassessing or reviewing it.

b. Policy Evidence
Please provide all supporting evidence for a program or policy in the form of URL links to webpages. If you indicated that this topic was a policy, you MUST provide a web link to the policy page.

1. **Smoking**

0: Smoking is permitted anywhere on campus, no smoking cessation programs or initiatives.

1: Encouraged smoking cessation. Smoking permitted in specific areas (i.e. away from buildings), smoking cessation pledge and programs.

2: A smoking policy can be located online. (NA)

NA This topic does not apply to this campus or this campus population.

2. **Alcohol**

0: There are no safe drinking initiatives or resources online.

1: Campus follows federal drinking laws; Safe, legal and moderate drinking is encouraged on campus webpages.

2: Campus has an alcohol policy; a safe alcohol education program might be mentioned in the body of the policy. NA

3. **Health Education NOT for Credit**

0: No health education resources/programs online.

1: Health education resources available online; Might be campus specific; health pledge (i.e. American Heart Association's "My heart. My life.")

2: A health education policy is online that requires non-mandatory health education programs to implemented online/on-campus. NA

4. **Nutrition Education NOT for Credit**

0: No nutrition education resources/programs online.

1: Nutrition education resources available online; Might be campus specific (i.e. healthy snack ideas on campus); might be general (i.e. MyPlate information)

2: A nutrition education policy is online that requires non-mandatory nutrition education programs to implemented online/on-campus. NA

5. **Physical Education NOT for Credit**

0: No physical education resources/programs online.

1: Physical education resources available online; Might be campus specific (i.e. walking routes on campus); might be general (i.e. American Heart Association Physical activity recommendations)

2: A physical education policy is online that requires non-mandatory physical education programs to implemented online/on-campus. NA

6. **Health Promotion (forms of media such as magazines, social media accounts, etc.)**

0: There are no forms of health promotion online.

1: Campus health blogs; mention of campus health magazine or digital download available.

2: A health promotion policy is online. Required health promotion by a department on-campus/online (i.e. weekly health newsletter blasts for all employees or students) NA

7. Campus Health Fair

0: No evidence of a campus health fair or promotion of a community health fair.

1:Campus advertises a local health fair; Campus has its own health fair.

2:A health fair policy is online that requires a health fair on campus or promotion of community health fair for campus members to attend. NA

8. Campus Health Screenings

0: No evidence of a campus health screening or promotion of one in the local community.

1: Campus advertises a local health screening; Campus has its own health screening; Campus encourages for members to seek health screenings on their own (i.e. blood pressure machine at health/grocery stores)

2: A health screening policy is online that requires a health screening on campus or promotion of community health screening for campus members to attend. NA

9. Chronic Disease Education

0: No evidence of chronic disease education online.

1: Campus chronic disease programs; off-campus chronic disease education program info; online chronic disease management education/materials.

2: Chronic disease policy that requires chronic disease promotion on campus; individuals with a specified chronic disease to seek education; etc. NA

10. Healthy Habit Challenges

0: No evidence of healthy challenges or rewards or incentives for healthy behaviors.

1: Campus encourages members to take a national health challenges. (i.e. AHA walking challenge); department healthy challenge programs; or campus wide healthy habit challenge program (i.e. Join campus in walking 10,000 steps/day)

2: Health habit challenge policy that requires campus (i.e. rec services department) to promote or implement healthy behavior challenges. Might specific if incentives need to be included. NA

11. Health Education Course Credit Requirement

0: There is no health education credit requirement or health education courses for credit offered on campus. (Do not include health education courses for specific programs i.e. "Advanced Exercise Physiology")

1:Health education courses available and intended for general campus population. (i.e. "How to Lead a Healthy Lifestyle") Health education course credit is NOT required for all undergraduate students.

2: Health education credit requirement. The policy states that "X" credits must be completed to graduate. NA

12. Nutrition education Course Credit Requirement

0: Do not include nutrition education courses for specific programs i.e. "Medical Nutrition Therapy"

1: i.e. "Introduction to Nutrition."

2: Nutrition education credit requirement. The policy states that "X" credits must be completed to graduate. NA

13. Physical Activity Education Course Credit Requirement

0: Do not include physical education courses for specific programs i.e. "Teaching Elementary Physical Education."

1: i.e. "Spinning" or "Intro to Running.") Physical education course credit is NOT required for all undergraduate students.

2: Physical education credit requirement. The policy states that "X" credits must be completed to graduate. NA

14. Physical Activity During Work Hours

0: No evidence that physical activity during work is promoted or permitted for employees.

1: No evidence that physical activity during work is promoted or permitted for employees.

2: There is a policy online that states employees should partake in physical activity during working hours to reduce sedentary time. NA

15. Active Environment (bike lanes, stairwells and sidewalks)

0: No online walking/biking routes are referenced online. No pictures parks/sidewalks on campus. No "take the stairs" initiative/program.

1: Walking/biking routes are referenced online; Pictures of on campus or nearby parks; A "take the stairs" initiative/program is promoted.

2: Policy regarding active environment and facility maintenance for both safety AND use of bike lanes, etc. for health purposes. NA

16. Closed Campus

0: Campus is open; No permit or pass is required to drive or park on campus.

1: Campus requires a pass/permit for driving/parking. There might be a policy that states restricted on campus driving is intended to limit traffic for safety purposes.

2:Campus policy states that campus completely closed. No parking/driving for general campus population. (excludes handicap, deliveries, etc.) NA

17. Sustainable Transportation

0: No evidence of sustainable transportation programs.

1: Sustainable transportation resources online; campus car pooling program; Zip cars; Bus pass reimbursements.

2: Policy in regards to sustainable transportation. Alternative transportation resources/incentives included in the text.

18. Campus Health and Wellness Department

0: No campus departments related to health and wellness

1: No campus central health/wellness department. Separate campus departments that collaborate. (i.e. Food services-Nutrition and Office of Health Promotion-mental health)

2: Policy that requires a single overarching department that is intended to promote to general health and wellness for campus population.

18a. Check all professionals whom are employed through the health/wellness department and/or university.

Medical Doctor, Registered Nurse , Nurse Practitioner , Registered Dietitian/Registered Dietitian Nutritionist , Employee with MPH , Certified sports specialist or expert (i.e. CPT), Specialty Medical Doctors (i.e. gynecologist), Psychologist

19. Healthy Campus Fundraising

0: No fundraising policy or promotion of healthy fundraisers.

1: Campus might promote healthy fundraisers (i.e. ideas for healthy fundraising article). Campus fundraising policy might exist (i.e. no fundraising, employees can't fundraise, or gambling fundraisers.)

2: Policy that limits/restricts fundraisers. (i.e. no alcohol) Might be required to be obesity-prevention related (i.e. no candy sales)

20. Healthy Employee Insurance Premium

0: Campus does not offer health insurance.

1: Campus encourages employees to enroll in health insurance; not required; there might be an initiative to keep insurance rates low; insurance incentives (i.e. $50 gift card for annual physical)

2: Requires all employees submit proof of insurance or enroll in campus insurance plan.

21. The food service department on this campus is

Contracted (i.e. Chartwells)

Independently run by the campus

21a. Healthy Food Options - procured, served, marketed and strategically placed

0: "Nutrition" not mentioned on the food service webpage

1: Nutrition information available online; Might not be campus specific (i.e. MyPlate); campus might have a healthy food pledge.

2: Policy requires healthy food options. Nutrition standards for healthy food (i.e. minimum % of healthy food options procured/served, etc.)

99: This campus does not serve food.

22. Nutrient Minimum / Maximum Standards

0: No nutrient information about food served on campus can be located.

1: Specific nutrient minimums or maximums standards; (i.e. trans fat free campus) All evidence is “standard practice” NOT a policy.

2: Campus has specific nutrient standards policy. Defines specific nutrient standards (i.e. Hot food will not exceed 500 mg sodium per serving)

99: This campus does not serve food.

23. Healthy Food Labels and Point-of-Purchase Nutrition Information

0: No evidence of a healthy food labeling program (i.e. Guiding Stars)

1: Healthy food option labeling or nutrition and ingredient labeling at point-of-purchase. Nutrition information is listed on menu boards if it is required by the state or county. (i.e. Starbucks on campus property)

2: Campus has a healthy food option labeling program and nutrient and ingredient labeling policy. Required nutrition information for food served on campus is required to be available.

99: This campus does not serve food.

24. Food Taxes and Healthy Food Subsidies

Is the campus located in a state/county with a food tax or healthy food subsidy policy?

Please provide a URL link to the state/county policy.

24a. Campus Food Taxes and Healthy Food Subsidies

0: No food tax or food subsidy on this campus.
1: Written statement/pledge to keep healthy food prices low to compete with unhealthy food options.
2: Food tax or food subsidy policy. The policy is NOT a federal, state or county policy. Policy defines the tax or subsidy (i.e all soda will be an addition 10 cents/bottle)
99: This campus does not serve or sell any food.

25. Designated Eating Environments and Mindful Eating
0: No designated places to eat on this campus (i.e. café or lunch room); No “lunch break” policy; No mindful eating programs.
1: Designated places to eat on campus; no “lunch break policy;” Mindful eating seminars/programs for population.
2: Designated places to eat on this campus; employees are required to take a lunch break; mindful eating programs might not be mentioned in the policy.

26. Local and Sustainable Food - Free-range, organic, local, fair-trade, etc.
0: No local or sustainable food is procured or marketed.
1: Local and sustainable food is mentioned; local vendors and farms are referenced; local food pledge/commitment
2: Policy requires that all food or specific foods come from within a specific mile radius; policy might state that during local seasons the campus will purchase from local farmers
99: This campus does not serve or sell any food.

27. Organic Waste Reduction and Disposal - recycling, composting, etc.
0: No sustainability initiatives.
1: Sustainability programs/initiatives in regard to food waste and food trash. (i.e. water used in dish room, drinking water, food compost, food donations, etc.)
2: Sustainability policy requires that all waste be composted; food service department must minimize water use; all water fountains will be installed in buildings to reduce water bottle purchases, etc.

28. Does this campus have a nearby local farmers market? (<10 miles from campus)
(Google search: {*city name*} farmers market)
Yes / No

29. Local Food Access On-Campus - campus farmers market, Community Supported Agriculture, and campus gardens
0: No local food access program
1: Student organization or club might run a CSA pickup on campus; campus garden; department plans/hosts a farmers market; bus from campus to local farmers market runs once/week, etc.
2: Campus has a local food access policy. It might define specific local food access initiatives and programs. Funding/department in charge should be stated in policy.

30. Does this campus have a campus garden that grows food?

Yes /No

31. On-Campus Housing

0: No on-campus housing for students.

1: On-campus housing available but not required. Students might be encouraged to live on campus for the first two years.

2: Policy states required number of years/class standing that students must live on campus..

99: This topic does NOT apply to this campus population.

32. Can students choose to eat on- or off-campus?

Yes / No

33. Dining Hall Contract

0: There is no evidence of dining hall contracts or meal plans available on this campus. All food is purchased independently.

1: There is a meal plan policy for campus members who wish to purchase one. The dining hall contract is optional. (ex. 1 meal/day)

2: This campus has a dining contract policy that requires students living on-campus to purchase a meal plan. Students living off-campus and employees can purchase an optional meal plan.

99: There are no dining halls or food courts on this campus.

34. Food Security Initiative

0: No food security resources referenced.

1: Resources for federal food assistance programs (SNAP or WIC); Campus food bank or weekly free meal; Campus office can assists in SNAP application.

2: Food security policy that requires a department to assist campus population in requiring means to purchase food, food, or enrolling in a food assistance program.
