# Supplementary material for: Development and Validation of the Policies, Opportunities, Initiatives and Notable Topics (POINTS) Audit for Campuses and Worksites
Source: Int J Environ Res Public Health. 2019 Mar 4;16(5):778. doi: 10.3390/ijerph16050778 (PMC6427413; doi:10.3390/ijerph16050778)
Supplement: Supplementary file 1 [file ijerph-16-00778-s001.zip › S2 HCI questions.pdf]

S2: Healthier Campus Initiative Survey Questions

1. Offer wellness meals at every meal in dining venues across campus.
2. Offer a variety of fruits, vegetables and 100% whole grain products in dining venues across campus.
3. Offer only a total number of fried foods in each dining hall that does not exceed the total number of platforms/stations in dining venues across campus.
4. Offer and identify as healthier at point of presentation desserts that have ≤150 calories as served.
5. Ensure the percentage of healthier beverage purchases (in dollars) is a minimum of sixty percent (60%) of total beverage purchases (in dollars).
6. Identify food and beverage items using one (1) of the following strategies: Label food and beverage items offered with calories per serving at the point of presentation OR / designate healthier food and beverage options using a healthy icon at the point o
7. Implement a comprehensive, strategic product placement/merchandising program/policy to encourage healthier food consumption.
8. Offer a comparable plant-based food option at platforms serving meat.
9. Implement a local food procurement program (e.g., Real Food Challenge) that increases procurement of local and sustainable foods.
10. Offer tray-less dining as the default system in dining venues.
11. Provide healthier vending options on campus by ensuring that a minimum of fifty percent (50%) of vending machines offer only healthier food and beverage products OR fifty percent (50%) of each vending machine content is healthier food and beverage product.
12. Provide healthier catering services on campus by developing a healthier catering menu that incorporates a variety of fruits, vegetables, 100% whole grain products, and no more than two (2) fried items.
13. Ensure the percentage of healthier beverage purchases (in dollars) is a minimum of sixty percent (60%) of total beverage purchases (in dollars) for catering.
14. Make free water available in all dining venues and all educational/physical activity facilities.
15. Make available Registered Dietitian Nutritionists (RDNs) for personal nutrition assessments and counseling to all students.
16. Provide marked walking routes on campus, at least two (2) miles in length, that have distance markers at regular intervals. Ensure a route map is made available to individuals on campus.
17. Post signage at each designated or marked crosswalk on campus requiring cars to stop for pedestrians.
18. Provide at least one (1) bicycle parking space on campus for every fifteen (15) individuals on campus.
19. Offer a bicycle share/rental program and/or a subsidized bicycle purchase program for all students.
20. Provide designated bicycle lanes on major roads and/or offer off-street bicycle paths throughout campus.
21. Implement a bicycle and pedestrian accommodation policy, (e.g., Complete Streets) and/or participate in a national bicycle or pedestrian recognition program (e.g., Bicycle Friendly University).
22. Implement a campus-wide program/policy that incentivizes the use of public or campus provided transportation (e.g., subsidized public transportation).

23. Provide, without a user fee, sixteen (16) hours per day access to at least one (1) fitness/recreation center for all students.
24. Dedicate at least seventy-five percent (75%) of the primary campus recreation center floor space to areas for physical activity.
25. Offer a minimum of forty (40) diverse non-competitive and competitive sports, group fitness, and intramural or informal recreation opportunities each academic year. Count each type of fitness opportunity as one, regardless of gender designation or level o
26. Offer, without a user fee, a minimum of one (1) monthly "how to" or beginner physical activity/ movement class that introduces students to new activities.
27. Offer at least one (1) organized and facilitated fifteen (15) minute physical activity/movement opportunity break on each school day.
28. Offer, without a user fee, both fitness/recreation center orientation during the first semester for all incoming students and one (1) fitness assessment to all students each academic year.
29. Provide at least one outdoor running/walking track that is open and available for use to individuals on campus and the community for at least three (3) hours per day.
30. Provide an outdoor fitness system.
31. Offer at least one (1) free, organized and facilitated, outdoor physical activity/movement opportunity each week.
32. Offer a rental outdoor recreation equipment program for students.
33. Offer at least twenty (20) outdoor recreation clinics and/or trips during each academic year.
34. Make available certified personal trainers for all students.
35. Implement an integrated, comprehensive wellness program for individuals on campus, that is provided annually that includes student, faculty, administrative and staff representatives, and meets at least quarterly. Activities hosted by the wellness committee
36. Implement a mandatory health and wellness education online module to be completed by all incoming first year or transfer students, upon registering with the college or university.
37. Implement a program/policy that identifies students who may be food insecure and provides options on campus.
38. Implement a program/policy that supports and accommodates breastfeeding for mothers on campus.
39. Implement a service-learning program available to all students that focuses on food and nutrition, physical activity/movement and/or coaching.
40. Offer a rewards-on-benefits structured program that gives insurance premium discounts and/or rebates to individuals on campus who participate in a wellness program designated by the health insurer on campus.
41. Offer in-person, non-academic cooking skills classes that are available to all students and incorporate fruits and vegetables.
